# Prognostic significance of a systemic inflammatory response in patients receiving first-line palliative chemotherapy for recurred or metastatic gastric cancer

**DOI:** 10.1186/1471-2407-11-489

**Published:** 2011-11-21

**Authors:** Jun-Eul Hwang, Ha-Na Kim, Dae-Eun Kim, Hyun-Jung Choi, Sung-Hoon Jung, Hyun-Jeong Shim, Woo-Kyun Bae, Eu-Chang Hwang, Sang-Hee Cho, Ik-Joo Chung

**Affiliations:** 1 Department of Internal Medicine, Division of Hemato-oncology, Chonnam National University Medical School, Gwangju, South Korea; 2 Department of Laboratory Medicine, Chonnam National University Medical School, Gwangju, South Korea; 3 Department of Urology, Chonnam National University Medical School, Gwangju, South Korea; 4 Department of Internal Medicine, Chonnam National University Hwasun Hospital, Jeollanamdo 519-809, South Korea

## Abstract

**Background:**

There is increasing evidence that the presence of an ongoing systemic inflammatory response is associated with poor prognosis in patients with advanced cancers. We evaluated the relationships between clinical status, laboratory factors and progression free survival (PFS), and overall survival (OS) in patients with recurrent or metastatic gastric cancer receiving first-line palliative chemotherapy.

**Methods:**

We reviewed 402 patients with advanced gastric adenocarcinoma who received first-line palliative chemotherapy from June 2004 and December 2009. Various chemotherapy regimens were used. Eastern Cooperative Oncology Group performance status (ECOG PS), C-reactive protein (CRP), albumin, Glasgow prognostic score (GPS), and clinical factors were recorded immediately prior to first-line chemotherapy. Patients with both an elevated CRP (>1.0 mg/dL) and hypoalbuminemia (<3.5 mg/dL) were assigned a GPS of 2. Patients in whom only one of these biochemical abnormalities was present were assigned a GPS of 1, and patients with a normal CRP and albumin were assigned a score of 0. To evaluate the factors that affected PFS and OS, univariate and multivariate analyses were performed.

**Results:**

According to multivariate analysis, the factors independently associated with PFS were ECOG PS (HR 1.37, 95% CI 1.02-1.84, *P *= 0.035), bone metastasis (HR 1.74, 95% CI 1.14-2.65, *P *= 0.009), and CRP elevation (HR 1.64, 95% CI 1.28-2.09, *P *= 0.001). The factors independently associated with OS were ECOG PS (HR 1.33, 95% CI 1.01-1.76, *P *= 0.037), bone metastasis (HR 1.61, 95% CI 1.08-2.39, *P *= 0.017), and GPS ≥ 1 (HR 1.76, 95% CI 1.41-2.19, *P *= 0.001).

**Conclusions:**

The results of this study showed that the presence of a systemic inflammatory response as evidenced by the CRP, GPS was significantly associated with shorter PFS and OS in patients with recurrent or metastatic gastric cancer receiving first-line palliative chemotherapy. Bone metastasis and GPS were very useful indicator for survival in patients with recurrent or metastatic gastric cancer receiving palliative chemotherapy.

## Background

Recurred or metastatic gastric cancer has a very poor prognosis, but chemotherapy can improve survival and possibly provide significant palliation of symptoms. Despite the recently reported benefits of chemotherapy, the 5-year survival rate for recurred or metastatic gastric cancer remains at ~ 5-20% [1-3]. Despite an often short and poor overall survival, there is marked heterogeneity in the duration of survival among patients. Therefore, there have been continuing efforts to investigate the prognostic factors related to survival [4,5].

C-reactive protein (CRP) is an acute phase protein which is synthesized by hepatocytes and;its levels in the serum increase during inflammatory diseases [6]. Cancer growth and resultant invasion induce local tissue damage, disturb local homeostasis, and cause systemic acute phase responses. Although the exact mechanism by which systemic inflammation arises in cancer patients remains to be clarified, it is generally accepted that cancer associated inflammation is modulated by cancer cells, host stromal cells, and their interactions [7].

There is increasing evidence that the presence of a systemic inflammatory response, as evidenced by elevated concentrations of CRP, is a prognostic factor in patients with advanced cancer [8-10]. High CRP levels are common in patients with advanced disease, because advanced cancer is often associated with an inflammatory response [7].

The Glasgow prognostic score (GPS) was introduced as a useful predictor for survival in patients with cancer by Forrest in 2003, and consists of the combination of 2 values, CRP and albumin [4,11]. It has been reported that GPS was associated with prognosis in various types of cancer including non small cell lung cancer, gastric cancer, colorectal cancer, pancreatic cancer, and breast cancer. Nearly all studies evaluated the prognostic value of preoperative CRP in resettable tumors [12]. Clinical factors such as liver metastasis, carcinomatosis peritonei, and bone metastasis are easily identifiable and imply that a patient has an advanced cancer. In this study, we evaluated the relationships between carcinomatosis peritonei, liver metastasis, bone metastasis, ECOG PS, albumin, CRP, GPS, and progression free survival (PFS), and overall survival (OS) in patients with recurrent or metastatic gastric cancer receiving first-line palliative chemotherapy.

## Methods

### Patients

We evaluated patients with advanced gastric cancer who had received palliative chemotherapy between June 2004 and December 2009 at Chonnam National University Hwasun Hospital (Gwangju, Korea). Patients were staged using a combination of endoscopy, computed tomography (CT) scan of the chest and abdomen, and additionally, positron emission tomography or bone scan when clinically indicated.

The criteria for case inclusion were as follows: (1) histologically confirmed gastric adenocarcinoma, (2) no prior chemotherapy or radiotherapy except for adjuvant treatment, (3) presence of metastatic disease, and (4) availability of clinical data at the initiation of therapy and follow-up. Of the 532 patients screened, 402 fulfilled the inclusion criteria and were enrolled in this retrospective analysis.

ECOG PS was evaluated according to the Eastern Cooperative Oncology Group criteria. The clinical tumor response was assessed radiologically by CT scanning after every 2 or 3 courses of chemotherapy according to the Response Evaluation Criteria in Solid Tumors (RECIST version 1.0) and clinically based on control of symptoms

Chemotherapy regimens had included a variety of agents such as taxane, irinotecan, cisplatin, oxaliplatin, 5-FU, S-1, and capecitabine. Oral fluoropyrimidines such as S-1 and capecitabine are replacing infusional 5-FU, and doublet chemotherapy regimens were most commonly used.

This study was approved by the institutional review board of Chonnam National University Medical School Research Institution (2011-109).

### Measurement of serum CRP

Routine laboratory measurements of CRP and albumin were carried out one day before the first cycle of chemotherapy. The limit of detection of the CRP assay was < 0.03 mg/dL, with the upper limit of normal values being < 1.0 mg/dL. Serum CRP was measured by latex turbidimetric immunoassay using a HITACHI 7600 (Hitachi, Tokyo, Japan). The coefficients of variation for these methods, over the range of measurements, were < 5%, as established by routine quality control.

The GPS was derived as previously described [4,11]. Patients with both an elevated CRP (> 1.0 mg/dL) and hypoalbuminemia (< 3.5 mg/dL) were assigned a score of 2. Patients in whom only one of these biochemical abnormalities was present were assigned of 1. Patients in whom neither of these abnormalities was present were assigned a score of 0.

### Statistics

The SPSS software package, version 17.0 (SPSS Inc, Chicago, IL, USA) was used for statistical analysis. The Kaplan-Meier analysis was applied to assess factors affecting overall survival (OS) and progression free survival (PFS): (1) OS was defined as the time from the first date of the first-line chemotherapy or to death from any cause or the last follow-up visit (2) PFS was defined as the time from the first date of the first-line chemotherapy to disease progression. Factors included in the univariate analysis using Kaplan-Meier methods were age, gender, liver metastasis, peritoneal metastasis, bone metastasis, albumin, CRP, ECOG PS, and GPS. Among the factors, those with *P *< 0.2 were selected and included in the multivariate regression analysis using the Cox proportional hazards regression model, which was performed to achieve an adjusted hazard ratio (HR) to determine prognostic factors for OS and PFS. *P *< 0.05 was considered significant for all analyses.

## Results

This study included a total of 402 patients who received first-line chemotherapy in the Department of Oncology at Chonnam National University Hwasun Hospital (Gwangju, Republic of Korea). We collected follow-up patient data from the cancer registry. All data were prospectively recorded and only the survival data was updated at the time of analyses. The median follow-up time was 11.4 months and ranged from 1.1 months to 58.5 months. The baseline clinical characteristics of the patients at the start of first-line chemotherapy are shown in Table 1 and Table 2. The median age of the patients was 59 years, with a range of 19-80 years. A total of 203 patients (72.9%) were male, and 73 patients (48.2%) had an ECOG PS of 2-3. A total of 125 patients (31.1%) had liver metastases, and 159 patients (39.6%), and 28 patients (7.0%) had peritoneal metastases and bone metastases, respectively. A total of 140 patients (34.9%) had an elevated CRP concentration (> 1 mg/dL), and 77 patients (19.2%) had hypoalbuminemia (< 3.5 mg/dL).

**Table 1:** Univariate analysis of clinical factors and GPS for progression free survival (*n *= 402)

	Patients (%)	mPFS months (95% CI)	*P*-value
Age			

<60	203 (50.5)	4.3 (3.8-4.7)	0.840
	
≥ 60	199 (49.5)	4.5 (3.9-5.1)	

Gender			

Male	293 (72.9)	4.5 (3.9-5.0)	0.381
	
Female	109 (27.1)	4.3 (3.6-4.9)	

Liver Mets			

Yes	125 (31.1)	4.1 (3.6-4.5)	0.243
	
No	277 (68.9)	4.6 (3.9-5.2)	

Peritoneal Mets			

Yes	159 (39.6)	4.3 (3.8-4.8)	0.128
	
No	243 (60.4)	4.6 (3.9-5.2)	

Bone Mets			

Yes	28 (7.0)	3.9 (2.4-5.3)	0.032
	
No	374 (93.0)	4.5 (4.0-5.0)	

Albumin			

<3.5	77 (19.2)	3.4 (2.2-4.5)	0.013
	
≥ 3.5	325 (80.8)	4.6 (4.1-5.2)	

CRP			

≤ 1	262 (65.1)	5.3 (4.5-6.0)	0.001
	
>1	140 (34.9)	3.4 (2.7-4.1)	

ECOG PS			

0-1	329 (81.8)	4.6 (4.0-5.1)	0.002
	
≥ 2	73 (48.2)	3.0 (2.0-3.9)	

GPS			

0	238 (59.2)	5.5 (4.8-6.2)	0.001
	
1	111 (27.6)	3.4 (2.7-4.1)	
	
2	53 (13.2)	3.3 (2.0-4.7)	

mPFS: median progression free survival, Mets: Metastasis, CRP: C-reactive protein, ECOG PS: Eastern Cooperative Oncology Group, performance status, GPS: Glascow prognostic score

**Table 2:** Univariate analysis of clinical factors and GPS for overall survival (*n *= 402)

	Patients (%)	mOS months (95% CI)	*P*-value
Age			

<60	203 (50.5)	11.6 (10.1-13.2)	0.808
	
≥ 60	199 (49.5)	12.0 (9.9-14.1)	

Gender			

Male	293 (72.9)	11.7 (10.1-13.3)	0.952
	
Female	109 (27.1)	12.1 (10.6-13.6)	

Liver Mets			

Yes	125 (31.1)	11.4 (8.5-14.3)	0.352
	
No	277 (68.9)	12.0 (10.7-13.3)	

Peritoneal Mets			

Yes	159 (39.6)	9.9 (8.4-11.5)	0.023
	
No	243 (60.4)	13.0 (11.2-14.8)	

Bone Mets			

Yes	28 (7.0)	8.2 (7.1-9.3)	0.117
	
No	374 (93.0)	12.1 (10.9-13.2)	

Albumin			

<3.5	77 (19.2)	7.7 (5.9-9.5)	0.001
	
≥ 3.5	325 (80.8)	12.4 (10.7-14.2)	

CRP			

≤ 1	262 (64.9)	14.8 (12.7-17.0)	0.001
	
>1	140 (34.8)	8.7 (7.3-10.2)	

ECOG PS			

0-1	329 (81.8)	12.4 (10.9-13.8)	0.003
	
≥ 2	73 (48.2)	8.7 (7.3-10.0)	

GPS			

0	238 (59.2)	15.3 (13.1-17.5)	0.001
	
1	111 (27.6)	8.9 (7.2-10.7)	
	
2	53 (13.2)	7.6 (4.9-10.2)	

mOS: median overall survival, Mets: metastasis, CRP: C-reactive protein, ECOG PS: Eastern Cooperative Oncology Group, performance status, GPS: Glascow prognostic score

The most commonly used first-line chemotherapy regimens were taxanes and cisplatin (*n *= 191, 47.5%). The specific chemotherapy regimens are shown in Table 3. A complete response to first-line chemotherapy was achieved in 25 patients (6.2%), and a partial response was achieved in 120 patients (29.9%), giving an overall response rate of 36.1% and a disease control rate of 70.9%. The results of chemotherapy are shown in Table 4. The median progression free survival was 4.4 months (95% CI 4.018-4.782), and the median overall survival was 11.8 months (95% CI 10.727-12.873).

**Table 3:** Regimens of chemotherapy

Regimen	No. of patients	%
Taxane/Cisplatin	191	47.5

Taxane/Cisplatin/5-FU	94	23.4

Irinotecan/5-FU	9	2.2

Oxaliplatin/5-FU	52	12.9

5-FU (oral or infusional fluoropyrimidines)/Cisplatin	21	5.2

Oral fluoropyrimidine	35	8.7

Total	402	100.0

**Table 4:** Response to chemotherapy (*n *= 402)

Response	No of Patient	%
CR	25	6.2

PR	120	29.9

SD	140	34.8

PD	103	25.6

ORR (CR + PR)	145	36.1

DCR (CR + PR + SD)	285	70.9

NE	14	3.5

Total	402	100.0

CR: complete response, PR: partial response, SD: stable disease, PD: progressive disease, ORR: overall response rate, DCR: disease control rate, NE: not evaluable

The univariate analysis demonstrated that 5 clinical factors were significantly associated with a shorter PFS; these factors included bone metastases, albumin < 3.5 mg/dL, CRP > 1 mg/dL, PS ≥ 2, GPS ≥ 1 (Table 1). The univariate analysis also demonstrated that 5 clinical factors were significantly associated with a shorter OS; these factors included peritoneal metastasis, albumin < 3.5 mg/dL, CRP >1 mg/dL, PS ≥ 2, and GPS ≥ 1 (Table 2).

**Figure 1:**
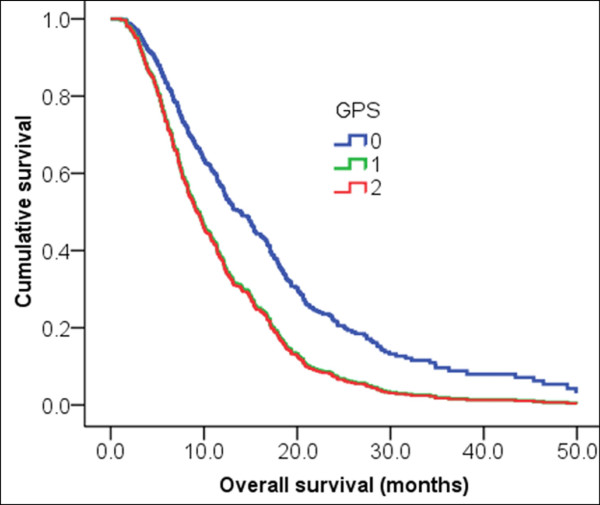
**GPS was an independent poor prognostic factor for OS in recurred or metastatic gastric cancer patients receiving first-line palliative chemotherapy (GPS = 1, HR 1.75 95% CI 1.37-2.26, *P *= 0.001; GPS = 2, HR 1.79, 95% CI 1.29-2.47, *P *= 0.001)**.

Cox multivariate analysis for PFS and OS was performed. Among the clinical factors, those with *P *< 0.2 were selected and included in the multivariate regression analysis using the Cox proportional hazards regression model. The result of the analysis identified the independent prognostic factors for PFS and OS. The independent poor prognostic factors for PFS were PS ≥ 2 (HR 1.37, 95% CI 1.02-1.84; *P *= 0.035), CPR >1 mg/dL (HR 1.64 95% CI 1.28-2.09; *P *= 0.001), and bone metastasis (HR 1.74, 95% CI 1.14-2.65; *P *= 0.009). The independent prognostic factors for OS were PS ≥ 2 (HR 1.33, 95% CI 1.01-1.76; *P *= 0.037), GPS 1 (HR 1.75, 95% CI 1.37-2.26; *P *= 0.001), GPS 2 (HR 1.79, 95% CI 1.29-2.47; *P *= 0.001), and bone metastasis (HR 1.61 95% CI 1.08-2.39; *P *= 0.017) (Table 5). Figure 1 shows the survival curve for patients according to GPS.

**Table 5:** Progression free survival and overall survival in advanced gastric cancer patients receiving first-line chemotherapy (multivariate analysis)

Factors	Progression free survival	
	
	Hazard ratio (95% CI)	*P*-value
ECOG PS (≥ 2)	1.37 (1.02-1.84)	0.035

CRP (>1)	1.64 (1.28-2.09)	0.001

Bone metastasis	1.74 (1.14-2.65)	0.009

	Overall survival	
	
	Hazard ratio (95% CI)	*P*-value

ECOG PS (≥ 2)	1.33 (1.01-1.76)	0.037

GPS (1)	1.75 (1.37-2.26)	0.001

GPS (2)	1.79 (1.29-2.47)	0.001

Bone metastasis	1.61 (1.08-2.39)	0.017

ECOG PS: Eastern Cooperative Oncology Group, performance status, CRP: C-reactive protein

In this study, GPS 1 was most commonly found to be a result of an elevated CRP (87 of 111, 78.4%). Among the patients with GPS 1, the patients with increased CRP and normal albumin level had a better median progression survival and median overall survival than the patients with normal CRP and decreased albumin level; however, the difference was not statistically significant (Table 6).

**Table 6:** Patients with increased CRP and normal albumin level had a better median progression free survival and median overall survival than patients with normal CRP and decreased albumin level in the patients with GPS 1 (not statistically significant)

	Patients with increased CRP and normal albumin level (*n *= 87)	Patients with normal CRP and decreased albumin level (*n *= 24)	*P*-value
mPFS (months)	3.433	2.533	0.489
		
	(95% CI 2.648-4.219)	(95% CI 0.000-5.284)	

mOS (months)	8.9	8.7	0.915
		
	(95% CI 7.003-10.930)	(95% CI 5.779-11.621)	

mPFS: median progression free survival, mOS: median overall survival, CRP: C-reactive protein

## Discussion

CRP is a nonspecific but sensitive marker of systemic inflammatory response, and might be expressed in selected tumor cells [13]. The biological basis for the correlation between elevations in this general marker of systemic inflammation and disease risk and outcome are not completely understood. The liberation of multiple proinflammatory cytokines, including interleukin-1 (IL-1), IL-6, and tumor necrosis factor-α from the tumor environment eventually results in the induction of CRP synthesis from the liver and other tissues [14]. Based on many recent studies, it is now widely accepted that an elevated CRP value is a reliable indicator of poor prognosis for certain malignant tumors including melanoma, non-Hodgkin's lymphoma, colorectal, lung, prostate and ovary [12,15,16].

Several studies demonstrated that low albumin concentrations are significantly associated with poorer survival in patients with gastric cancer [17,18]. In patients with gastric cancer, it is recognized that there may be reduced caloric intake due to stenosis of the cardia or pylorus. However, several recent reports showed that the systemic inflammatory response plays a major role in the progressive nutritional and functional decline of patients with cancer [19]. Indeed, measurement of the systemic inflammatory response, particularly CRP, has been included in the definition of cancer cachexia, together with weight loss and reduced calorie intake [20]. It thus appears that the development of hypoalbuminemia is secondary to an ongoing systemic inflammatory response and poor cancer specific survival is secondary to the systemic inflammatory response in patients with gastric cancer [21]. GPS can thus reflect both the presence of a systemic inflammatory response and the progressive nutritional decline of the patient with advanced cancer. In this study, GPS 1 was most commonly associated with increased CRP (87 of 111, 78.4%). Our results also demonstrated that increased CRP was associated with shorter PFS, and a GPS ≥ 1 was associated with shorter OS in advanced gastric cancer in palliative chemotherapy settings.

In gastric cancers, almost all studies evaluated the prognostic value of the preoperative CRP in resettable tumors [12]. In a recent study of 204 patients who underwent curative resection of gastric carcinoma, preoperative CRP elevations were found to be independently predictive of shorter overall survival [22]. To our knowledge, only 2 studies demonstrated the relationship between GPS and cancer specific survival in unresectable gastric cancer [4,5]. These studies showed that GPS was a significant independent predictor of cancer survival in unresectable gastroesophageal cancer. However, these studies also included esophageal cancer and diverse treatment modalities such as chemotherapy, radiotherapy, and endoscopic laser were used for palliative treatment. In our study, we only enrolled patients receiving first-line palliative chemotherapy for gastric adenocarcinoma. We excluded gastroesophageal junction cancer, because the underlying pathophysiology of cancers of the gastroesophageal junction and the corresponding therapeutic approach differ substantially from those for gastric cancer proper [23].

Although gastric cancer is thought to spread more commonly to the peritoneum or liver than to bone, the reported incidence of skeletal lesions varies between 1 and 45% [24]. The presence of bone metastasis has consistently been associated with poor prognosis in studies of Korean gastric cancer patients. In their report analyzing 1,455 metastatic gastric cancer patients who received first-line chemotherapy, Lee et al. reported that the presence of bone metastasis was an independent poor prognostic factor [18]. In our study, bone metastasis was also independently associated with poor prognosis (median OS 8.2 vs. 12.1 months, bone metastasis (+) vs. (-), *P *= 0.017).

The mechanism by which a systemic inflammatory response might influence cancer survival is not clear. It may be that an elevated systemic inflammatory response is associated with a poor local immune response to the tumor and therefore increased lymph node spread and metastasis [21]. Also, the presence of a systemic inflammatory response and the associated nutritional decline may influences tolerance and compliance with active treatment [25,26]. Andreyev et al. reported that the poorer outcome of chemotherapy in advanced gastrointestinal cancer patients with weight loss appears to be as a result of receiving less chemotherapy due to toxicity rather than poorer tumor response [27]. In this study, among the patients with GPS 1, the patients with normal CRP and decreased albumin level (*n *= 24) had a tendency to have a poorer median progression free survival and median overall survival than the patients with increased CRP and normal albumin level (*n *= 87). In this group of the patients, decreased albumin level might be associated with the chronic nutritional decline and poor ECOG PS.

## Conclusions

The presence of a systemic inflammatory response as evidenced by the CRP and GPS, appears to be a useful indicator of prognosis in patients receiving first-line chemotherapy for metastatic gastric cancer. Poor performance status and bone metastasis were also associated with poor prognosis. CRP, GPS, and bone metastasis are easily identifiable and objective clinical parameters. These parameters could facilitate the individual patient risk assessment and thus, a more appropriate prediction of survival for each patient.

## Competing interests

The authors declare that they have no competing interests.

## Authors' contributions

JEH is a main author. DEK, WKB, HJS, SHJ and SHC performed the chemotherapy for patients and revised the manuscript. ECH made special contributions to the statistical analysis. IJC conceived of the study, and approved the final manuscript. All authors read and approved the final manuscript.

## Pre-publication history

The pre-publication history for this paper can be accessed here:

http://www.biomedcentral.com/1471-2407/11/489/prepub
